# Focal myocardial fibrosis assessed by late gadolinium enhancement cardiovascular magnetic resonance in children and adolescents with dilated cardiomyopathy

**DOI:** 10.1186/s12968-015-0142-0

**Published:** 2015-05-15

**Authors:** Heiner Latus, Kerstin Gummel, Karin Klingel, Axel Moysich, Markus Khalil, Nona Mazhari, Juergen Bauer, Reinhard Kandolf, Dietmar Schranz, Christian Apitz

**Affiliations:** Pediatric Heart Centre, University Children’s Hospital Giessen, Feulgenstr. 12, D-35392 Giessen, Germany; Department of Molecular Pathology, University Hospital Tübingen, Tübingen, Germany

**Keywords:** Childhood dilated cardiomyopathy, Myocardial fibrosis, Late gadolinium enhancement, Reverse ventricular remodelling

## Abstract

**Background:**

Different patterns of late gadolinium enhancement (LGE) including mid-wall fibrosis using cardiovascular magnetic resonance (CMR) have been reported in adult patients presenting with non-ischemic dilated cardiomyopathy (DCM). In these studies, LGE was associated with pronounced LV remodelling and predicted adverse cardiac outcomes. Accordingly, the purpose of our study was to determine the presence and patterns of LGE in children and adolescents with DCM.

**Methods:**

Patients <18 years of age presenting with severe congestive heart failure who were admitted for evaluation of heart transplantation at our centre underwent CMR examination which consisted of ventricular functional analysis and assessment of LGE for detection of myocardial fibrosis. Ischemic DCM was excluded by coronary angiography, and right ventricular endomyocardial biopsies ruled out acute myocarditis.

**Results:**

Thirty-one patients (mean age 2.1 ± 4.2 years) with severe LV dilatation (mean indexed LVEDV 136 ± 48 ml/m^2^) and LV dysfunction (mean LV-EF 23 ± 8%) were examined. LGE was detected in 5 of the 31 patients (16%) appearing in various patterns characterized as mid-wall (*n* = 1), focal patchy (*n* = 1), RV insertion site (*n* = 1) and transmural (*n* = 2). Based on histopathological analysis, 4 of the 5 LGE positive patients had lymphocytic myocarditis, whereas one patient was diagnosed with idiopathic DCM.

**Conclusions:**

In children and adolescents with DCM, focal histologically proven myocardial fibrosis is rarely detected by LGE CMR despite marked LV dilatation and severely depressed LV function. LGE occurred in various patterns and mostly in patients with inflammatory cardiomyopathy. It remains unclear whether myocardial fibrosis in childhood DCM reflects different endogenous repair mechanisms that enable favourable reverse remodelling. Larger trials are needed to assess the prognostic implications of LGE in childhood DCM.

## Background

Dilated cardiomyopathy (DCM) is the most common form of cardiomyopathy in children and is characterized by a dilated and globally hypocontractile left ventricle. Childhood DCM is a heterogenous disease associated with high morbidity and mortality [1–3], especially in patients with idiopathic DCM [4]. Patients frequently develop severe congestive heart failure. If pharmacological treatment fails, patients are listed for cardiac transplantation (HTx) or must receive mechanical circulatory support. However, the clinical course can be variable and some patients remain stable or even show reverse remodelling with normalization of LV dimensions and function [5, 6]. Although specific risk factors for adverse outcome have been found in children with DCM [2, 4, 7, 8], additional parameters for reliable risk stratification and prediction of a reverse ventricular remodelling are required to guide management of these patients.

Cardiovascular magnetic resonance (CMR) has gained an important role in the diagnosis of patients presenting with systolic heart failure. Late gadolinium enhancement (LGE) imaging enables identification and quantification of focal macroscopic myocardial fibrosis. In adult patients with non-ischemic DCM, various studies demonstrated not only a diagnostic but also prognostic role of LGE CMR [9–12]. In these studies, different patterns of local myocardial fibrosis including the typical pattern of “mid-wall” fibrosis assessed by LGE have been reported in up to 50% of adult patients with DCM [9–13].

CMR assessment of myocardial fibrosis has been reported to be useful in patients with congenital heart disease [14, 15] and cardiomyopathies [16], however, data about LGE imaging in children is limited. The purpose of our study was to determine whether biopsy-proven myocardial fibrosis can be assessed by LGE in children with DCM and whether its occurrence is related to the cause of DCM and in what pattern myocardial fibrosis occurs. Furthermore, the impact of myocardial fibrosis on reverse ventricular remodelling should be evaluated.

## Methods

### Study population

Patients under 18 years of age presenting with congestive heart failure who were admitted for evaluation of cardiac transplantation at our centre underwent CMR as part of our routine clinical examination. The diagnosis of DCM was made according to current guidelines: patients with a dilated left ventricle, defined by left ventricular end-diastolic dimension with a z-score of greater than 2 and an ejection fraction below a z-score of -2 were included in the study. Patients with congenital cardiac disease including abnormal origin of the coronary arteries or Kawasaki disease, neuromuscular or immunologic disease, familial cardiomyopathy, endocrine disorders, metabolic or mitochondrial disease and clinical evidence of acute myocarditis (based on the results of the histopathological/immunohistological analysis of the endomyocardial biopsies) were excluded.

Clinical data were retrospectively obtained from hospital medical records including date of birth, gender, cause of DCM and age at CMR evaluation. Findings of the CMR studies were extracted from the routine clinical reports. Laboratory findings of B-type natriuretic peptide (BNP) and troponin I (TNI) at initial admission were also assessed. Clinical follow-up data of the patients included information about further treatment (i.e. medical heart failure therapy, pulmonary artery banding, left ventricular mechanical support, cardiac transplantation) and survival. Patients who were treated by medical heart failure therapy, follow-up data on echocardiographic data (LV dimension and function) were also assessed for the evaluation of reverse ventricular remodelling (defined as normalisation of LV size and function, i.e. z-score of LVEDD and fractional shortening between +2 to -2 [5]). The study protocol was approved by the local ethics committee and all patients or parents of the patients gave written informed consent for participation in the study.

### Cardiac catheterization protocol, histopathological/immunohistological analysis of endomyocardial biopsies and detection of viral genomes

Cardiac catheterization was performed in all patients. Endomyocardial biopsies were performed in 30 of the 31 patients. In each patient two to three biopsies were taken from the RV interventricular septum using a transcatheter bioptome. In one patient there was no myocardial biopsy available, because myocardial perforation occurred at the first attempt of biopsy sampling with subsequent need for emergency surgery.

Histopathological analysis was performed as previously described [17, 18]. Endomyocardial biopsies were stained with Masson’s trichrome as well as Giemsa and examined by light microscopy. Inflammatory cells were quantified with immunohistochemical staining using CD3 (T cells), CD68 (macrophages) and HLA-DR-α to assess HLA class II expression in professional antigen-presenting immune cells. Nested (RT-) polymerase chain reaction was performed for the detection of enteroviruses (including coxsackieviruses group A and B and echoviruses), parvovirus B19 (PVB19), adenoviruses, human cytomegalovirus, Epstein-Barr virus, and human herpes virus type 6 (HHV6). The heart was considered to be inflamed after immunohistochemical detection of focal or diffuse mononuclear infiltrates with >14 leukocytes per 1 mm^2^ (CD3^+^ T lymphocytes and/or CD68^+^ macrophages) in the myocardium, in addition to enhanced expression of HLA class II molecules.

### CMR protocol

All CMR studies were performed on a 3-T system (Verio, Siemens, Erlangen, Germany). Images were acquired with two sixteen-elements phased array coils. Sedation was applied in most of the patients.

#### Cine CMR

Images were acquired in supine position with two sixteen-elements phased array coils. Sedation was applied in younger patients when considered necessary. The CMR protocol included a stack of short-axis slices from the base of the heart to the apex using cine steady-state free precession (SSFP) with breath-hold or gradient echo (GE) sequences in free-breathing technique when patients were sedated. Data acquired during breath-hold were assessed with the following sequence parameters: TR 48 ms, TE 1.5 ms, flip angle 60°, slice thickness 6 mm, in plane image resolution 1.3 mm × 1.3 mm × 6.0 mm, temporal resolution 25-40 phases. In measurements during free-breathing, the sequence parameters were as follows: TR 56 ms, TE 2.5 ms, flip angle 12°, slice thickness 5 mm, in plane image resolution 1.4 mm × 1.4 mm × 5.0 mm. End-diastolic (maximal) and end-systolic (minimal) volumes, stroke volumes (SV) and ejection fractions (EF) for the RV and LV were calculated by dedicated software (ARGUS, Siemens, Erlangen, Germany) after the RV and LV endocardial borders were traced manually at end-systole and end-diastole. All parameters were adjusted to body surface area (BSA).

#### Late gadolinium enhancement CMR

Before administration of gadolinium, renal function parameters, i.e. glomerular filtration rate, as well as creatinine levels were assessed to exclude renal dysfunction. LGE was assessed after intravenous injection of gadopentetate dimeglumine (Magnevist, Bayer, Leverkusen, Germany) at a dose of 0.15 mmol/kg of body weight by using a two-dimensional inversion-recovery segmented gradient echo CMR sequence in the cardiac short and long-axis planes (slice thickness 5 mm, field of view 300 mm). The inversion time was adjusted for optimal suppression of signal from normal myocardium and the images were obtained within 5-10 min after injection (inversion time approximately 250-350 ms). All LGE images were interpreted accordingly to the American Heart Association 17-segment model. The patterns of LGE were classified as subendocardial-based, transmural, mid-wall striae, mid-wall patchy, subepicardial, RV insertion point and diffuse. Extent of LGE was assessed using certified software (cmr^42^, Circle Cardiovascular Imaging Inc., Calgary, Canada).

## Statistical analysis

Continuous variables are presented as mean with standard deviation or median and range, as appropriate. Correlations were tested using linear regression analysis. Analysis was performed using GraphPad statistical software package (San Diego, California, USA). A *p* value ≤0.05 was considered statistically significant.

## Results

### Patient population

Between 01/2009 and 05/2014 thirty-one patients (17 females, median age 7.0 (1-203) months, median weight 6.6 (3-57) kg) were included in the study. The majority of patients (*n* = 21, 68%) were less than one year of age (Table 1). Associated congenital heart disease included an atrial septal defect in one patient. On admission, 16 patients were in NYHA/Ross class IV, 13 patients in class III and 2 patients in class II. Mitral valve regurgitation on echocardiography was °1 in 16 patients, °2 in 12 patients and °3 in 3 patients. Mean levels of BNP were 1900 ± 2019 pg/ml and mean TNI levels were 0.18 ± 0.28 μg/l.

**Table 1:** Patient characteristics

Variable	Value
Patients, n	31
Male/Female	14/17
Height, cm	65 (53-161)
Weight, kg	6.6 (3-57)
BSA, m^2^	0.46 ± 0.33
Age at study, months	7 (1-203)
Age < 4 weeks, n (%)	3 (10)
Age 1-24 months, n (%)	21 (68)
Age 2-12 years, n (%)	5 (16)
Age 12-18 years, n (%)	2 (6)
Cause of DCM	
Unknown, n (%)	15 (48)
Chronic lymphocytic myocarditis, n (%)	16 (52)
Detection of virus by endomyocardial biopsy, n (%)	11 (35)
HHV6, n	6
PVB19, n	3
Enteroviruses, n	2
Blood testing	
Troponin positive, n (%)	17 (55)
Tropnin, μg/l	0.18 ± 0.28
BNP, pg/ml	1900 ± 2019
NYHA-class, I/II/III/IV, n	0/2/13/16
Medication	
ACE-I/ARB, n (%)	24 (77)
Beta-blocker, n (%)	24 (77)
Diuretics, n (%)	27 (87)
Digoxin, n (%)	13 (42)
Antiarrhytmic, n (%)	5 (16)
Inotropcis, n (%)	16 (52)

*DCM* dilated cardiomyopathy, *BSA* Body surface area, *PVB19* parvovirus B19, *HHV6,* human herpes virus type 6, *BNP* b-type natriuretic peptide, *NYHA* New York Heart Association, *ACE-I* angiotensin-converting inhibitors, *ARB* angiotensin receptor blocker; Data are presented as mean standard deviation (SD) or median and range, as appropriate

### Endomyocardial biopsies

According to the results of the histopathological/immunohistological analysis of the endomyocardial biopsies, 15 patients (48%) revealed DCM without and 16 patients (52%) with inflammation (DCMi, inflammatory cardiomyopathy) corresponding to the diagnosis of chronic myocarditis (Table 1). Positive viral genome testing was reported in 11 patients with detection of HHV6 in 6, PVB in 3 and enteroviruses in 2 patients. Histopathological analysis was not available in one patient (who showed no signs of acute myocarditis and had negative viral serology testing).

### CMR findings

Mean heart rate at CMR was 115 ± 22 bpm. Mean indexed (i) LVEDV was 136 ± 48 ml/m^2^ and mean LVESVi 106 ± 42 ml/m^2^ resulting in an LVSVi of 30 ± 14 ml/m^2^ (Table 2). Mean LV ejection fraction (EF) was 23 ± 8%. Mean RVEDVi was 65 ± 36 ml/m^2^, mean RVESVi 36 ± 30 ml/m^2^ and RVSVi 29 ± 11 ml/m^2^. Mean RV-EF was 50 ± 13% but ranging from 12 to 66%.

**Table 2:** CMR findings

Variable	Value
Heart rate, bpm	115 ± 22
LVEDVi, ml/m^2^	136 ± 48
LVESVi, ml/m^2^	106 ± 42
LVSVi, ml/m^2^	30 ± 14
LVEF, %	23 ± 8
RVEDVi, ml/m^2^	65 ± 36
RVESVi, ml/m^2^	36 ± 30
RVSVi, ml/m^2^	29 ± 11
RVEF, %	50 ± 13
LGE positive, n (%)	5 (16)
LGE, % of LV mass	6.1 ± 5.3

bpm, beats per minute; LV, left ventricle; RV, right ventricle; i, indexed; EDV, enddiastolic volume; ESV, endsystolic volume; SV, stroke volume;EF, ejection fraction; LGE, late gadolinium enhancement; Data are presented as mean standard deviation (SD)

**Fig. 1:**
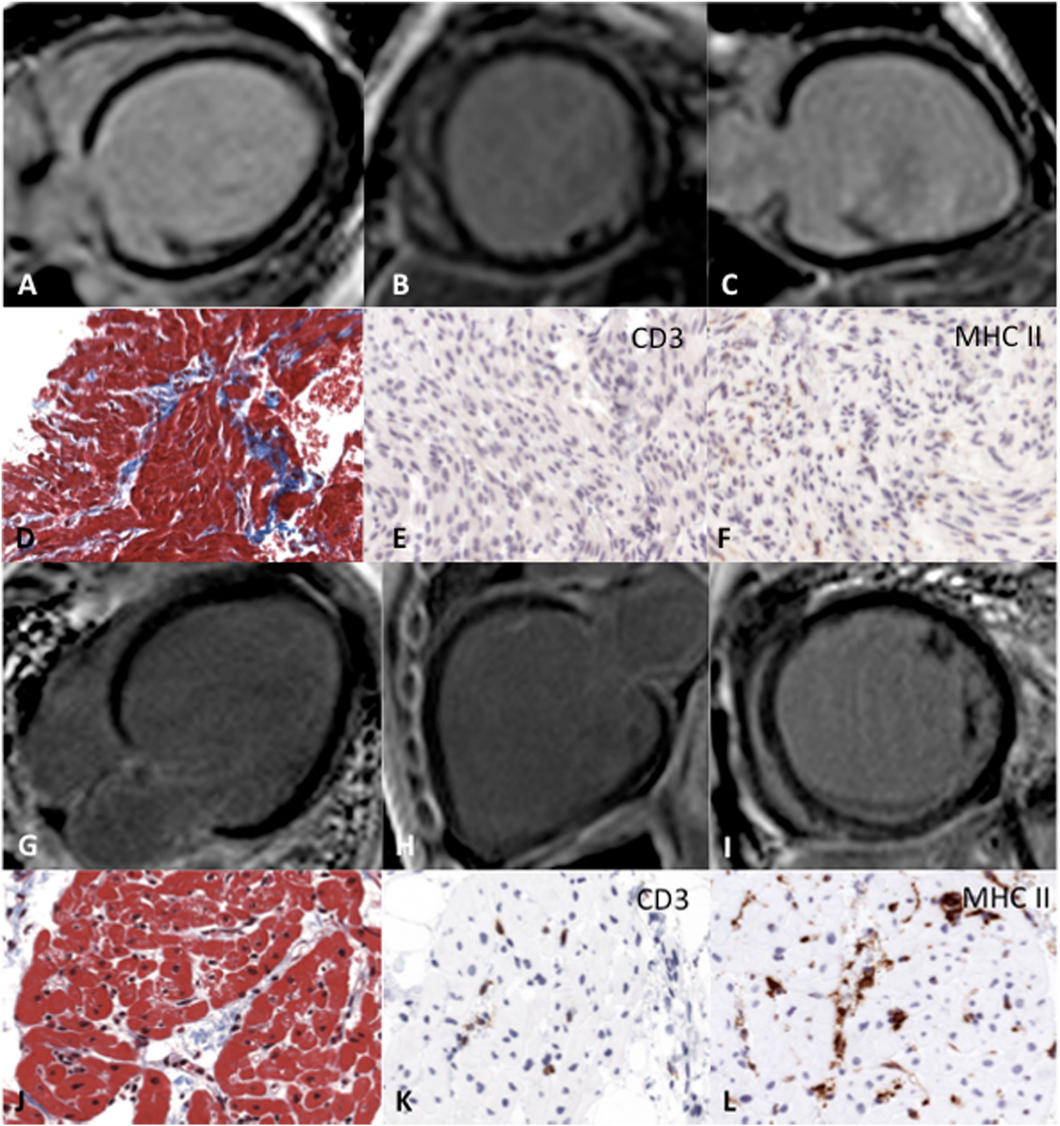
Example of two patients with DCM and no signs of myocardial fibrosis assessed by LGE CMR and the corresponding histopathological and immunohistochemical findings CMR images of a 6 month-old patient with idiopathic DCM (**A-C**) and of a 4 month-old patient with DCM with lymphocytic myocarditis (**G-I**). Masson’s trichrome staining (**D** and **J**) reveals in both patients lesions characterized by interstitial fibrosis (blue) and the presence of degenerated myocytes. While in the patient with inflammatory cardiomyopathy the number of T lymphocytes (CD3) (**K**) and expression of HLA II class molecules on macrophages were increased (**L**), the uninflamed heart revealed no T cells (**E**) or enhanced expression of HLA class II molecules (**F**)

**Fig. 2:**
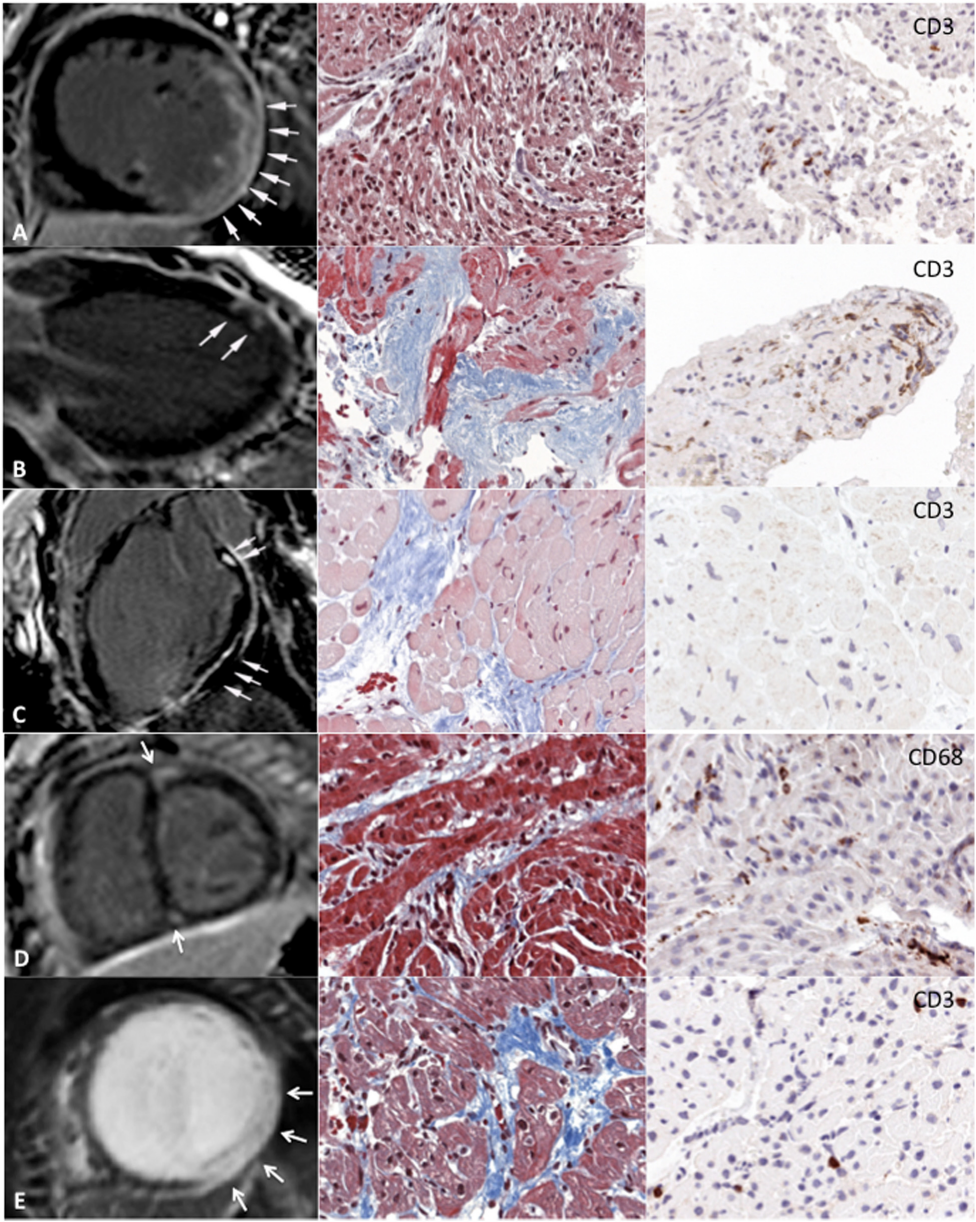
Results of CMR (left column) and histopathology/immunohistology of the five patients in whom LGE was detected. Various patterns of LGE were found (transmural LGE in A and E; focal patchy LGE in C; midwall LGE in C; RV insertion LGE in D). The column in the middle shows the Masson’s trichrome staining illustrating the presence of fibrosis (*blue*) while the right column displays immunohistochemical analysis reveals detection of T cells (CD3) or macrophages (CD68). Inflammatory cardiomyopathy was found in four patients (A,B,D,E) whereas the heart of patient C showed no signs of inflammation

LGE was detected in 5 of the 31 patients (16%) appearing in various patterns characterized as focal patchy (1 patient, aged 12 months with lymphocytic myocarditis), transmural (2 patients, aged 5 and 7 months, both lymphocytic myocarditis), RV insertion fibrosis (1 patient aged 1 month with lymphocytic myocarditis) and classic ‘mid-wall’ (1 patient, aged 16 years, idiopathic DCM) (Figs. 1 and 2). The mean extent of LGE of moycardial mass was 6.1 ± 5.3% (range from 1.7 to 14.7%).

#### Correlations

**Fig. 3:**
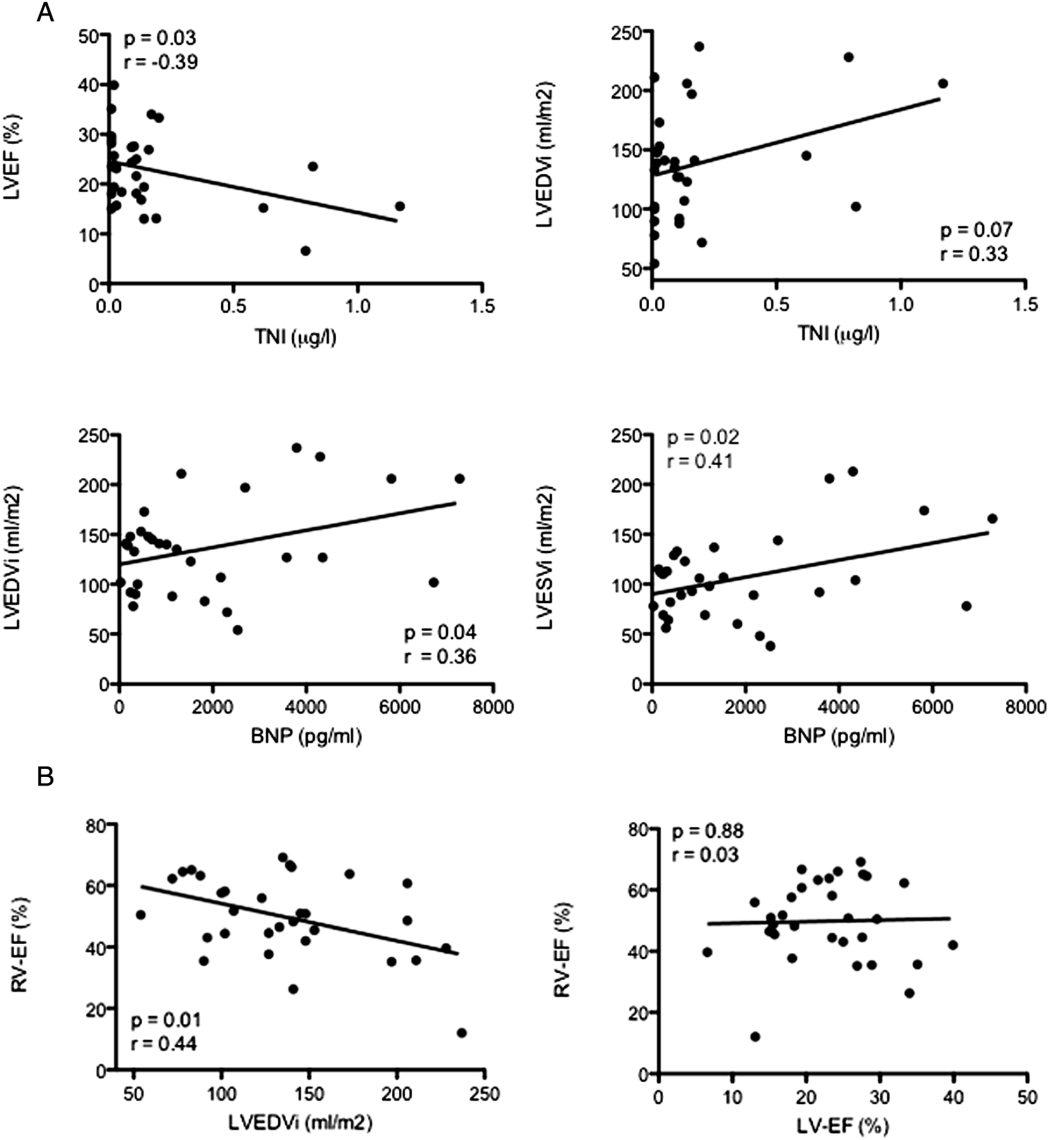
Relationships between cardiac dimensions (LVEDVi, indexed left ventricular enddiastolic volume) and levels of troponin I (TNI) and B-type natriuretic peptide (BNP). A significant correlation between left ventricular cardiac dimensions and right ventricular function (RV EF, right ventricular ejection fraction) was found whereas left and right ventricular function were found to be unrelated

Levels of troponin I were related to the degree of LV dysfunction (r = -0.39, *p* = 0.03) and showed a trend with increasing LV diameter (r = 0.33, *p* = 0.07) (Fig. 3). Levels of B-type natriuretic peptide correlated significantly with LVEDVi (r = 0.36, p = 0.04) and LVESVi (r = 0.44, *p* = 0.02) but not with LV-EF (r = -0.24, *p* = 0.19). RV parameters were not related to BNP levels. Significant correlations were found between LVEDVi and RV-EF (r = -0.44, *p* = 0.01), whereas LVEDVi showed no relationship with RVEDVi (r = 0.20, *p* = 0.24). No relationship was found between LV-EF and RV-EF (r = 0.03, *p* = 0.88).

### Follow-up and occurrence of reverse ventricular remodelling

**Fig. 4:**
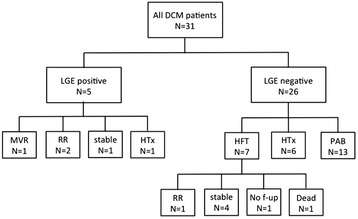
Follow-up including outcome and occurrence of reverse remodelling in the study population. *DCM,* dilated cardiomyopathy; *LGE,* late gadolinium enhancement; *MVR,* mitral valve replacement; *RR,* reverse remodelling; *HFT,* medical heart failure therapy; *HTx,* cardiac transplantation; *PAB,* pulmonary artery banding; *f-up,* follow-up

Of the 26 LGE negative patients 13 underwent pulmonary artery banding (PAB) (Fig. 4) as a rescue therapy combined with medical heart failure therapy as previously reported by our group [19]. This intervention was offered for patients with preserved right ventricular function and informed consent was obtained from the parents for this compassionate use treatment. Another 6 patients underwent cardiac transplantation while 7 patients received only medical heart failure therapy. Of these latter patients, one patient showed spontaneous reverse remodelling, four patients were clinically stable with unchanged levels of LVEDD values and LV function while one patient died and another one was lost to follow-up. Of the five LGE positive patients, none died or had to undergo mechanical circulatory support. One of the patients with transmural LGE underwent mitral valve replacement due to progressive severe mitral valve insufficiency (probably related to myocardial scarring) leading to further atrial and ventricular enlargement while the other patient underwent cardiac transplantation. In the two other patients (one with focal patchy and one with RV insertion fibrosis) cardiac dimensions and function recovered to normal values after 3 and 22 months indicating spontaneous reverse remodelling. The 16 year-old patient with mid-wall fibrosis declined implantation of a cardioverter-defibrillator due to episodes of ventricular tachycardia while clinical status and cardiac dimensions remained stable.

## Discussion

This study demonstrated that in young children and adolescents with dilated cardiomyopathy

1. The detection of histologically proven focal myocardial fibrosis by LGE is uncommon (found only in 16%) although LV dilatation was marked and LV function severely depressed,
2. The majority of LGE positive patients had chronic myocarditis suggesting a relation between histological signs of inflammation and LGE,
3. Various patterns of LGE were found with transmural type of LGE being detected in two of the 5 patients, which is not typical for non-ischemic forms of DCM and might reflect embolic myocardial infarction.

The findings of our study are in contrast to previously reported CMR findings in adult patients with non-ischemic DCM where LGE was detected more frequently (in up to 50% of patients) and was associated with larger left ventricles, a higher degree of impaired function, elevated biomarkers and arrhythmias resulting in worse overall outcome [9–12, 20]. Accordingly, the presence of focal LGE in non-ischemic DCM in adults is considered to be associated with contractile impairment that is thought to reflect the transition from compensated to decompensated state representing the endpoint of adverse remodelling with irreversible ventricular failure [9]. As the severity of LV dilatation and functional impairment in our study group was comparable to that reported in series of adult patients, the low rate of macroscopic fibrosis detected by LGE in our study is unexpected and the underlying reason for this finding entirely unknown.

In general, limited data exists about the pathogenesis of myocardial fibrosis in neonates, children and young adolescents with all types of cardiomyopathies including its assessment by LGE [16, 21–23]. Grosse-Wortmann and colleagues [24] reported 11 children and adolescents with DCM of various etiologies but found myocardial fibrosis by LGE only in one patient. Although the severity of LV dilatation and dysfunction was more pronounced in our study group, the prevalence of myocardial fibrosis by LGE did not increase. Furthermore, as reported by this group, RV dimensions and function differed widely among the patients illustrating that childhood DCM can present as a biventricular disease although no relationship between RV and LV dimensions was detected. However, the severity of LV dilatation negatively affected RV function probably due to adverse ventricular-ventricular interactions while BNP levels were solely related to LV dimensions.

Specific patterns of LGE have been reported in patients with ischemic and non-ischemic DCM linking the type of LGE to a certain form of cardiomyopathy. In non-ischemic DCM mid-wall striae of LGE is known to be the typical finding, although various patterns such as focal patchy, subepicardial and diffuse LGE have also been described [9–12, 25, 26]. Interestingly, the only patient in our study with mid-wall LGE had idiopathic DCM and was the oldest patient in the study cohort (17 years of age) while a midwall focal patchy pattern of LGE and RV insertion site fibrosis were seen in one patient with lymphocytic myocarditis, respectively. It has been described that myocarditic infiltrations can persist after the acute phase of myocarditis, but tend to regress over time during healing [17, 27–29]. Conversely, a transmural pattern of fibrosis is not typically for myocarditis or idiopathic DCM, rather than for ischemic heart disease, but was found in two patients with myocarditis. These scars could be related to an embolic event that subsequently caused myocardial infarction or might be associated with interstitial expansion due to inflammation and/or edema as these patients were diagnosed for inflammatory cardiomyopathy. However, discrimination of ischemic from non-ischemic lesions is crucial in these patients and thus might influence therapeutic strategy and prognosis.

The underlying mechanisms for the development of fibrotic changes in non-ischemic cardiomyopathies are related to different processes. Myocardial injury can be the result of different pathogens and toxins causing inflammation, individual genetic susceptibility, abnormal modulation of the immune system, permanent adrenergic activation and metabolic dysregulation [30–32]. Furthermore, increased wall stress due to LV enlargement can lead to microvascular ischemia with subsequent myocyte necrosis which might explain why troponin levels were found to be related to LV dimension and function in our population. The resulting focal areas of replacement fibrosis are thought to be the basis for the detection of LGE by CMR although LGE can also be the result of other forms of interstitial expansion. Although good agreement between areas of LGE and the pathological location of macroscopic fibrosis on autopsy has been reported [10, 11], the study by Schalla and colleagues showed that the presence of focal LGE in adult DCM patients was more related to microscopic findings of inflammation rather than fibrosis in endomyocardial biopsy [33]. This might explain why the majority (4 of the 5 patients) of our LGE positive patients were found to have lymphocytic myocarditis. Otherwise, the low detection rate of LGE in our population does not imply that myocardial fibrosis is absent in childhood DCM. Indeed, only a few histopathological studies exist about childhood DCM, but those also revealed fibrosis as a cardinal feature [34, 35]. This is in accordance with our findings, where analysis of endomyocardial biopsies revealed a substantial degree of fibrosis in endomyocardial biopsies, both in patients with and without LGE on CMR.

This suggests that the possible pathogenic factors and mechanisms leading to the phenotype of LV dilatation and dysfunction considerably vary in younger patients. Although speculative, the low incidence of myocardial fibrosis assessed by CMR might be related to differences in myocardial remodelling in DCM between children and adults. Nishikawa et al. analysed endomyocardial biopsies and found differences in the distribution of microscopic patterns of fibrosis, revealing a higher myocarditic index and a higher incidence of bizarre myocardial hypertrophy as well as discrete ultrastructural differences between adults and children with DCM [34, 36] suggesting that factors and mechanisms causing myocardial damage may differ between children and adults. Furthermore, differences in myocardial matrix structure and function [37] as well as in adrenergic receptor stimulation [38] have been described, which might have an impact on response to medical therapy thereby influencing ventricular remodelling and prognosis.

Decision making whether cardiac transplantation is needed in the individual child with DCM is crucial but is hampered by a substantial rate of spontaneous recovery of ventricular dimension and function that has been described both in adult and childhood DCM [3, 5, 6, 39, 40]. A recent multicentre study by Everitt et al. demonstrated a 2-year cumulative incidence of recovery of normal LV size and function in 22% of patients [5]. One explanation for this phenomenon might be related to our findings of a less remodelled myocardium which inherits a higher chance for recovery. These results can be supported by the study of Masci et al. who found in adult patients with idiopathic DCM, that the absence of LGE was a strong independent predictor of LV reverse remodelling at 2-years follow-up independent of LV size and function [13]. Nabeta and colleagues recently demonstrated that the estimation of fibrosis by baseline CMR-LGE is superior in predicting reverse remodelling and outcome than the estimation of fibrosis by baseline endomyocardial biopsy [41]. Due to the high number of patients in our study cohort who underwent pulmonary artery banding or cardiac transplantation, procedures that affect the natural history of this disease, only a minority of patients received exclusively medical heart failure therapy not allowing any further statistical analysis of the predictive value of LGE in children with DCM. However, detection of LGE seemed not to have an impact on the occurrence of reverse remodelling or outcome which demonstrates that CMR LGE may not be able to separate those patients with a high chance for recovery from those who will be affected by an aggravation in ventricular function with a subsequent higher risk for death and/or cardiac transplant. Whether myocardial fibrosis in childhood DCM is a factor for evaluating innovative treatment options also taking advantage of the suggested preserved cardiac regenerative capacity of the myocardium of young children will require further investigations [42–44].

### Study limitations

The small study population and the retrospective design of the study were accompanied by a selection bias towards patients presenting in an advanced stage of the disease who were referred for cardiac transplantation might not represent the entire population of children and adolescents with DCM. Therefore, the reported incidence of LGE as well as the observed rate of spontaneous recovery and outcome might differ in reality.

Compared to previous studies, we observed a higher incidence of patients with myocarditis which must be considered when interpreting the data. The reason for this might be related to the fact that other causes for DCM, especially hereditary forms, were excluded in order to analyse a homogenous patients cohort. While the underlying genetic causes for childhood DCM are increasingly recognized [45], routine genetic testing for mutations known to cause DCM is not performed at our institution which may have resulted in a certain selection bias.

Identification of LGE in very young patients may be limited by respiratory or motion artifacts related to spontaneous breathing under sedation. Furthermore, spatial resolution of the CMR sequence might be too low to detect small areas of local fibrosis in neonates and infants thereby underestimating its real prevalence. However, in a previous study in neonates and infants with ischemic DCM [21], LGE could be reliably assessed. Because no additional method besides LGE was used to confirm histologically proven areas of myocardial fibrosis thereby potentially missing important signs of adverse ventricular remodelling. Furthermore, the used LGE technique cannot detect perivascular and interstitial myocardial fibrosis that is frequently observed in non-ischemic DCM [46]. Further developments of T1 mapping sequences that enable reliable quantification of the extracellular space in younger patients may potentially provide further and earlier insights into adverse cardiac remodelling in pediatric heart failure.

## Conclusions

In children and adolescents with DCM, focal histologically proven myocardial fibrosis is rarely detected by LGE CMR despite marked LV dilatation and severely depressed LV function. LGE occurred in various patterns and mostly in patients with inflammatory cardiomyopathy. It remains unclear whether myocardial fibrosis in childhood DCM reflects different endogenous repair mechanisms that enable favourable reverse remodelling. Despite the small study population, our findings suggest that LGE may not be able to predict ventricular recovery or allowing reliable risk stratification as seen in adult patients. Nevertheless, the observed differences in incidence of myocardial fibrosis compared to adult patients may influence the therapeutic strategy in childhood DCM taking advantage of a less remodelled LV.

## Abbreviations

ACE-I: Angiotensin-converting inhibitors
ARB: Angiotensin receptor blocker
BNP: B-type natriuretic peptide
BSA: Body surface area
CD: Cluster of differentiation
CMR: Cardiovascular magnetic resonance
DCM: Dilated cardiomyopathy
EDV/ESV/SV: Enddiastolic/endsystolic/stroke volume
EF: Ejection fraction
HHV: Human herpes virus
HLA: Human leucocyte antigen
LGE: Late gadolinium enhancement
LVEDD: Left ventricular end-diastolic diameter
LV: Left ventricle
NYHA: New York Heart Association
PAB: Pulmonary artery banding
PVB 19: Parvovirus B19
RV: Right ventricle
TNI: Troponin I
